# Supplementary material for: Patterns of Intron Gain and Loss in Fungi
Source: PLoS Biol. 2004 Nov 30;2(12):e422. doi: 10.1371/journal.pbio.0020422 (PMC532390; doi:10.1371/journal.pbio.0020422)
Supplement: Table S1 — Also available at http://genes.mit.edu/NielsenEtAl/. (4.3 MB ZIP). [file pbio.0020422.st001.zip › NielsenEtAl/html/875.html]

AN0247.1.NCU02284.1.MG11039.1.FG00732.1


```
 CLUSTAL W (1.82) Multiple Sequence Alignments - Introns Inserted


Sequence 1: NCU02284.1	1062 aa
Sequence 2: MG11039.1	1030 aa
Sequence 3: FG00732.1	1068 aa
Sequence 4: AN0247.1	1076 aa
Alignment Length: 1090 aa
Number Identitical Residues: 627 aa
Alignment Score (without introns) 28343


MG11039.1 	------~----------------------------------~---MTQVGLKQNKSVLWA
NCU02284.1	MSQKVI2DSRIPALIRNGMQEKKRSFFVVVGDHSKEAIVHL1YYILSNANMKHNKSVLWA
FG00732.1 	MQRKAV2DSRIPALIQNGLQEKKRSFFVVVGDRSKDVIVRL~HYIMSQFDIKQNKSVLWA
AN0247.1  	MPRKAI~DSRIPALIQNSIQEKKRNFFVVVGDRAKDIIVNL~YHIRSQFDVKQNKSVLWA
          	   .   .:  .:   .. .... .     .. :..          :: .:*:*******

MG11039.1 	YKKQLLGFTS~HRRKRETKIKKEIKRGTREANDADPFELFLSLQNIRYVYYKETEKILGN
NCU02284.1	YKSKLLGFTS~HRKKRENKIKKEIKRGVREANSEDPFELFISLNDIRYVYYKETEKILGN
FG00732.1 	YKNKLLGFTS2HRKKREEKIKKEIKRGIREANTEDPFELFVSLHNIRYTYYKETDKILGQ
AN0247.1  	YKKDLLGFTS~HRKKREAKIKREVKRGIREPNEEDPFELFVTLNQIRYVYYKETEKILGN
          	**..****** **:*** ***:*:*** **.*  ******::*::***.*****:****:

MG11039.1 	TYGM0CILQDFESITPNILARTIETVEGGGMVILLLKGMSSLKQLYTMTMDVHSRYRTEA
NCU02284.1	TYGM~CILQDFEAMTPNILARTIETVEGGGLVILLLKGMNSLKQLYTLSMDVHSRYRTEA
FG00732.1 	TFGM~CILQDFEAITPNILARTIETVEGGGLVVLLLKGMNSLKQLYSLSMDVHSRYRTEA
AN0247.1  	TYGM~CILQDFEAITPNLLARTIETVEGGGTVIMLLKSMNSLKQLYTLSMDIHSRYRTEA
          	*:** *******::***:************ *::***.*.******:::**:********

MG11039.1 	YDDVVARFNERFILSLGSCESCLVVDDELNVLPISGGKNVKQLPEPESSEDAKTPAQKEL
NCU02284.1	HDDVVARFNERFILSLGSCESCLVVDDELNVLPISGGKAVKALPRPTDDEEVN-PAKKEL
FG00732.1 	HDDVVARFNERFILSLGSCNSCLVIDDEMNVLPISGGKGVKKLP-PPDLDNAKTESQIEL
AN0247.1  	HDDVVARFNERFILSLGSCDSCLVIDDEMNVLPISGGKNVKPLPPPDETDGTSKGTKKEL
          	:******************:****:***:********* ** ** * . : ... :: **

MG11039.1 	DEVKDSLRETQPVGSLLQLARTNDQAKALLTFVDAISEKTLRNTVTLTAARGRGKSAAMG
NCU02284.1	EKIKESLEDTQPIGSLVQLARTTDQAKALLTFVDAIAEKTLRNTVTLTAARGRGKSAAMG
FG00732.1 	EAMKEQNEGRQPVGPLISLAKTVDQAKALMTFTDAIAEKTLRSTVTLTAARGRGKSAAMG
AN0247.1  	EKIKDDLAESQPVGSLVNLARTVDQAKALLTFVDAIAEKTLRSTVTLTAARGRGKSAALG
          	: :*:.    **:*.*:.**:* ******:**.***:*****.***************:*

MG11039.1 	VAIAAAVAYGYSNIFITSPSPENLKTLFEFVFKGLDALDYKDHADYSIMQSTNPDFNKAI
NCU02284.1	VAIAAAVAYGYSNIFITSPSPENLKTLFEFVFKGFDALDYKDHADYSIIQSTNPEFNKAI
FG00732.1 	VAVAAAVAYGYSNIFITSPSPENLKTLFEFVFKGFDELGYADHADYSIIQSTNPDFHKAI
AN0247.1  	VAIAAAIAYGYSNIFITSPSPENLKTLFEFIFKGFDALGYADHADYTILQSTNPDFNKAI
          	**:***:***********************:***:* *.* *****:*:*****:*:***

MG11039.1 	VRVNIHRNHRQTIQYIRPQDSHVLGQAELVVIDEAAAIPLPLVKKLMGPYLVFMASTING
NCU02284.1	VRVNIHRNHRQTIQYIRPQDAHVLGQAELLVIDEAAAIPLPLVKKLMGPYLVFMASTISG
FG00732.1 	VRVNIHRQHRQTIQYIRPQDAHVLGQAELLVIDEAAAIPLPLVKKLMGPYLVFMASTING
AN0247.1  	VRVNIHRQHRQTIQYIQPQDAHVLGQAELLVIDEAAAIPLPLVRKLMGPYLVFMASTING
          	*******:********:***:********:*************:**************.*

MG11039.1 	YEGTGRSLSLKLIKQLREQSRAPVAGNNVDVEID-RSSGKAEKN--ATMAGARSLKEITL
NCU02284.1	YEGTGRSLSLKLIKQLREQSRT-GAGVKDAVEVD-RTSGKAAKDTETVFTGGRSLKEITL
FG00732.1 	YEGTGRSLSLKLIKQLRDQSRTASTAGEGMEIAD-RSTGKTSKT--EEFQAGRKLREITL
AN0247.1  	YEGTGRSLSLKLIQQLREQSRGGAKIAGDDTDVASRATGKIEKNS-DKGLGGRSLREITL
          	*************:***:***             :*::**  * :     ..*.*:****

MG11039.1 	SEPIRYGPGDAVEKWLNTLLCLDATLPKSKLNTQGCP0DPTQCELLQVNRDTLFSFHPVS
NCU02284.1	SEPIRYAQGDPVEKWLNTLLCLDATLPRSKLNTQGCP~DPSQCELLHVNRDTLFSFHPVS
FG00732.1 	SEPIRYAQGDAVEKWLNTVLCLDATLPKAKSSINGCP~DPTQCQLLNVNRDTLFSFHPVS
AN0247.1  	SEPIRYAPGDSVEKWLNRVLCLDATLPKSKMNTQGCP~HPSQCQLLQVNRDTLFSFHPVS
          	******. **.****** :********::* . :*** .*:**:**:*************

MG11039.1 	ERFLQQMVALYVASHYKNTPDDLQLMSDAPAHKLFVLTPPPSEN--SLPEPLCVIQVALE
NCU02284.1	EKFLQQMVALYVASHYKNSPNDLQLMSDAPAHELFVLTAPIVEG--RLPEPLCVIQVSLE
FG00732.1 	EKFLQQMVALYVASHYKNSPDDLQLMSDAPAHELFVLVPPVSENSARLPEPLCVIQVSLE
AN0247.1  	EKFLQQMMALYVASHYKNTPNDLQLMSDAPAHQLFVLVPPIDEDAAKLPEPLCVIQVALE
          	*:*****:**********:*:***********:****..*  *.:: **********:**

MG11039.1 	GKISRQSILNSLSRGQRPAGDLIPWLVSQQFQDEEFASLSGARVVRIATNPDYVSMGYGT
NCU02284.1	GKISKQSILNSLSRGSQPAGDLIPWLVSQQFQDDEFASLSGARVVRIATNPDYVSMGYGS
FG00732.1 	GKISRQSVLNSLSRGQRPNGDLIPWLVSQQFQDEDFASLSGARVVRIATNPEYVSMGYGT
AN0247.1  	GRISRQSVLNSLSRGQRAGGDLIPWLVSQQFQDEDFASLSGARIVRIATNPEYLSMGYGS
          	*:**:**:*******.:. **************::********:*******:*:*****:

MG11039.1 	RALELLVDFFEGKSLSLSEDVEDMVEKTMPRVTDAELSKLGAGSLLDDNIRVRDMNEMPP
NCU02284.1	RALQLLVDYYEGKFANLSEEAGTVVPKSAPRVTDAELAN---ASLFDD-IKVRDMNELPP
FG00732.1 	KALELLVDYYEGRFTSLSEDEDQVMEETMTRVTDAELAN---ANLLDDDIKVRDINKMPP
AN0247.1  	RALELLVDFFEGKFTDLSENIVDTQEE-MVRVTDEELAN---SNLLDDNIQVRDIRSMPP
          	:**:****::**:  .***:      :   **** **::   ..*:**.*:***:..:**

MG11039.1 	LFSYMHQCRPPPLDYVGVSYGLTQPLHKFWKRASFAPVYL0RQTANDLTGEHTCVMLRPL
NCU02284.1	LFAKLAERRPEPLEYIGVSYGLTQPLHKFWKRASFAPVYL~RQTPNDLTGEHTCVMLRPL
FG00732.1 	LFSKLSEKKPETLDYVGVSYGLTSPLHKFWKRASFSPVYL~RQTANDLTGEHTCVMLRPL
AN0247.1  	LFGKLSERRPDQLDYLGVSYGLTPALHKFWKRASFTPVYL~RQTPNDLTGEHSCVMLRTL
          	**. : : :*  *:*:******* .**********:**** ***.*******:*****.*

MG11039.1 	NT-TEDQSWLGAFTNDFHRRFLNLLSYQFREFPSVLSLSIDESAVSGAQTDSTNEPSPLT
NCU02284.1	GD-GTDPSWLGAFANDFHRRFLSLLSYKFREFPAILALTIEESASVGAKLDPSNEPTPLE
FG00732.1 	EN-SGDRSWLGAFSRDFQRRFLSLLSYQFRSFSPVTALSIEESANAGAKLDS-FELQPLT
AN0247.1  	STGSNDISWLGAFARDFHRRFISLLSYQFRTFASVLSLSIGESATAGAKLDPSLAPSPLT
          	  .  * ******:.**:***:.****:** *..: :*:* ***  **: *.:    ** 

MG11039.1 	KADLDRLLTPFDQKRLESYAKNLLDYHVILDMVPTLAHLYFTGALKSSIKLTGVQQAILL
NCU02284.1	KAELDKLLTPFDHKRLESYANGLLDYHVVLDLIPSIAQLFFEGRIRSNLQLSGLQQAILL
FG00732.1 	RSDLDIYMTPFDLKRLVSYSNNMLDYHVIIDLVPTIASLYFTGRLKGNVSLTGVQQSILL
AN0247.1  	KSDLDAAFSPFDLKRLDSYANNLLDYHVILDMVPIIATWYFSGRLAGKVNLSGVQQSILL
          	:::**  ::*** *** **::.:*****::*::* :*  :* * : ..:.*:*:**:***

MG11039.1 	AVGLQRKDIDVVSSELSLPSSQLLAMFIKIMRKITTHFETLVTQAVDAEMPKPKSIGVSR
NCU02284.1	AIGLQRKEIDAIAGELTLPSSQLLAIFIKIMRKISTHLASLVEGAIAKEMP--QQIGVSA
FG00732.1 	AIGLQHKDIEVISGEIGLPTSQLLAMFIKIMRKFAGHLNEVVTTAVDAELPKAERLGVSR
AN0247.1  	AIGLQRKSLDDVEKELSLPSSQLLAMFLKIVRKVSTHFRSLEESAVADTLP---AENVPA
          	*:***:*.:: :  *: **:*****:*:**:**.: *:  :   *:   :*     .*. 

MG11039.1 	ENALGAHDDEIVDNRFVPLAKGLGEELEEVGDEALKEYRLKQKELIDSLPLDQ2FEVEGG
NCU02284.1	ENASAAHDDEIIENNFVPLDQNLDDELAEGGDQAMKAIRAKQRELIDSLPLDQ~YEIDGG
FG00732.1 	EDADGAHDDEIVDERFVPLTTTLDEELEEGGDEALRELKKKQRELIDSLPLDQ2YEIEGD
AN0247.1  	TSAAADAHDEVVDERFKPLETSLDDELREGGEEINAEMREKQRALIDALPLDK2YEISNG
          	 .* .  .**::::.* **   *.:** * *::     : **: ***:****: :*:...

MG11039.1 	ASEWKEAEKAIAKAASKGETPAMTVSVKSQKTKRKAGQQTAAEVYEEAFGEKRHKKAKKS
NCU02284.1	DAEWKEAEKRVAKATKEGKS-NPLVSVKTKKRK-------AEPVYEEEKSEKRDRGDKKG
FG00732.1 	APAWEDAEKQVKSAAKEGKS-NPVVSVKSTKQKRKADQKTAAEVYEEEFGEK--KKNKKS
AN0247.1  	DAGWEEAEKQIRSGG------AATVSVKSSKQNKRKKGETAREIYEQEIESKRQKIIQKG
          	 . *::*** : ..          ****: * :     .:*  :**:   .*  :  :*.

MG11039.1 	KKEKGA--~---------
NCU02284.1	KKVRHDKR~K--------
FG00732.1 	KRSKKSD-~---------
AN0247.1  	TEGKKKKR2LIGYIQKLK
          	.. :  .    .  .. .
```
